# Supplementary material for: Stomatal and Non-Stomatal Leaf Traits for Enhanced Water Use Efficiency in Rice
Source: Biology (Basel). 2025 Jul 10;14(7):843. doi: 10.3390/biology14070843 (PMC12293053; doi:10.3390/biology14070843)
Supplement: Supplementary file 1 [file biology-14-00843-s001.zip › biology-3707211-supplementary.pdf]

Supplementary Table

Table S1. Comprehensive list of rice genes involved in (A) stomatal and (B) non-stomatal leaf traits affecting water use efficiency. The table includes genes spanning key functional categories including stomatal development and regulation, cuticular wax biosynthesis, leaf morphology, stress response pathways, and metabolic adaptations. Locus IDs correspond to the Rice Annotation Project and were verified via the SNP-Seek database from the International Rice Research Institute [149]. Directional trends in the WUE Association column indicate: ↑ = positive association with increased WUE (gene overexpression or enhanced function improves water use efficiency); ↓ = negative association with WUE (gene overexpression or enhanced function reduces water use efficiency); ± = variable or context-dependent association (effects depend on environmental conditions, genetic background, or developmental stage).

**A. Stomatal leaf Traits**			
**Gene**	**Locus ID**	**Role**	**WUE Association**
OsEP3	LOC_Os02g15950	Regulates stomatal guard cell development [150]<br>*ERECT PANICLE3*	↑ WUE<br>Optimises stomatal development for better water regulation
OsKAT1	LOC_Os01g55200	Regulates stomatal opening and closing by facilitating potassium ion flux, which directly affects stomatal movement and plant water regulation [151,152]<br>*K+ TRANSPORTER 1*	↑ WUE<br>improves stomatal control
OsSDD1	LOC_Os03g0143100	Mutations lead to higher stomatal density [13]<br>*STOMATAL DENSITY AND DISTRIBUTION*	↓ WUE<br>increases water loss via excess stomata
OsTMM	LOC_Os01g02060	Mutations can lead to irregular stomatal spacing, affecting gas exchange efficiency [13]<br>TOO MANY MOUTHS	↓ WUE<br>disrupts stomatal patterning
OsEPF1	LOC_Os04g0637300	Overexpress reduce stomatal density [38]<br>*EPIDERMAL PATTERNING FACTOR1*	↑ WUE<br>reduces transpiration
OsSPCH1	LOC_Os06g33450	Essential for the initiation of stomatal lineage, termination of meristemoid fate and the transition to guard mother cell (GMC) identity. Overexpression	↓ WUE<br>Overexpression results in increased water loss

 https://doi.org/10.3390/biology14070843

		expression leads to higher stomatal density [33, 153] *SPEECHLESS1*	
OsSPCH2	LOC_Os02g15760	alterations in stomatal size and density in stress conditions. Overexpression increases density [13,154] *SPEECHLESS2*	↓ WUE Overexpression results in increased water loss
OsMUTE	LOC_Os05g51820	Essential for the initiation of stomatal lineage, termination of meristemoid fate and the transition to GMC identity. Involves in the differentiation of stomatal precursor cells. It plays a critical role in determining stomatal size by regulating the development of guard cells [153]	↑ WUE ensures proper stomatal function
OsFAMA	LOC_Os05g50900	Essential for the initiation of stomatal lineage, termination of meristemoid fate and the transition to GMC identity [153]	↑ WUE Maintains correct stomatal identity for optimal function
OsFLP	LOC_Os07g43420	Regulates the orientation of GMC symmetrical division [153] *FOUR LIPS*	↑ WUE Regulates stomatal spacing for efficient gas exchange
**B. Non-stomatal leaf traits**			
**Gene**	**Locus ID**	**Role**	**WUE Association**
OSH43	LOC_Os03g56110 LOC_Os03g57560	Overexpression results in broader leaves, increased tiller number, and more flowers [92]. *ORYZA SATIVA HOMEOBOX43*	↑ WUE No direct evidence.
OsA-BCG9	LOC_Os04g44610	Mutations can lead to reduced cuticular wax content, resulting in increased sensitivity to drought and other environmental stresses [155]	↓ WUE Mutation increases drought sensitivity due to water loss
OsGL1-1	LOC_Os09g25850 LOC_Os09g25850	Increase leaf cuticular wax deposition and enhance drought tolerance [62] *GLOSSY1-1*	↑ WUE Enhances drought resistance
OsGL1-2	LOC_Os02g08230	Overexpression increases cuticular wax production and improves drought tolerance [156] *GLOSSY1-2*	↑ WUE Overexpression reduces water loss

OsWR1	LOC_Os02g10760	Overexpression increases the expression of genes involved in wax synthesis [157] *WAX SYNTHESIS REGULATORY GENE1*	↑ WUE<br>Overexpression reduces water loss
OsCutA1	LOC_Os10g23204	Overexpression increases cuticular wax production and improves drought tolerance, a promising gene for engineering rice plants with enhanced drought tolerance [61]	↑ WUE<br>Overexpression reduces water loss
YGL1	LOC_Os05g28200	Overexpression results in darker green leaves, increased chlorophyll content, and increased photosynthetic activity. This leads to improved growth and increased yields [158]<br>*YELLOW-GREEN LEAF1*	↑ WUE<br>Overexpression improves carbon assimilation efficiency
OsHB2	LOC_Os10g33960	Overexpression of the OsHB2 gene results in longer roots, broader leaves, and increased tolerance to drought and salinity stress [159]<br>*HOMOEOBOX1*	↑ WUE<br>Overexpression improves water capture and leaf area for photosynthesis
RCN1	LOC_Os11g05470	Inhibiting flowering transition and delaying heading under drought. Many rachis branches in the panicle and high yield [160]<br>*ROOTS CURL IN NAPHTHYLPHTHALAMIC ACID1*	± WUE<br>Delays maturity but may improve yield under drought
OsCKX2	LOC_Os01g10110	Produce larger leaves, more flowers, and higher yields [161]<br>*CYTOKININ OXIDASE/DEHYDROGENASE2*	↑ WUE<br>Improves resource allocation for higher yield
Gn1a	LOC_Os01g10110	Improves grain yield, resulting in more flowers and larger grains [162]<br>*GRAIN NUMBER 1a identical to CKX2*	↑ WUE<br>Increases yield potential under stress
NAL3	LOC_Os12g01120	Overexpression results in broader leaves, reduced tiller number, increased	↑ WUE

		lateral root development, and larger grains [163]<br>*NARROW LEAF3*	Overexpression enhances soil water access and light interception
NRL1	LOC_Os12g37190	Overexpression results in wider leaves, increased plant height, larger vascular bundles, stronger stems, and higher yields [92,164]<br>*NARROW AND ROLLED LEAF1*	↑ WUE<br>Overexpression improves plant robustness and water transport
NAL1	LOC_Os04g52479	Overexpression results in broader leaves, reduced tiller number, increased lateral root development, and larger grains [165]<br>*NARROW LEAF1*	↑ WUE<br>Improves growth under water-limited conditions
NAL7	LOC_Os03g06654	Increased expression could have wider leaves, taller plants, and higher yields [92,165]<br>*NARROW LEAF7*	↑ WUE<br>Enhances light interception and productivity
OsSPL14	LOC_Os08g39890	Regulates tiller number and panicle branching, which can also lead to increased grain yield [166]<br>*SQUAMOSA PROMOTOR BINDING PROTEIN-LIKE14*	↑ WUE<br>Enhances yield per unit water use
OsSPL9	LOC_Os05g33810	Increases grain number per panicle and yield, and regulates drought tolerance, which can improve rice performance in dry environments [167]<br>*SQUAMOSA PROMOTOR BINDING PROTEIN-LIKE9*	↑ WUE<br>Enhances productivity under drought stress
WSL1	LOC_Os06g39750	Involved in the biosynthesis of cuticular wax in rice leaves [168]<br>*WHITE STRIPED LEAF1*	↑ WUE<br>Reinforces leaf cuticle to reduce evaporation
OsMyb6	LOC_Os06g10350	Overexpression in rice improves drought and salinity tolerance [169]<br>*MYB-TKF6*	↑ WUE<br>Overexpression enhances stress response and water retention
OsPIP1;1	LOC_Os02g44630	facilitate osmotic water transport across membranes [170]	± WUE

		PLASMAMEMBRANE INTRINSIC PROTEIN1;1	Can improve water uptake or increase loss depending on conditions
OsPIP2;1	LOC_Os07g26690	contributes to water transport and is highly expressed in roots and leaves [170] *PLASMAMEMBRANE INTRINSIC PROTEIN1;1*	± WUE Context-dependent water transport regulation
OsNAC6	LOC_Os01g66120	Regulates stress responses, including drought tolerance, by modulating root architecture and other physiological traits. Overexpressing transgenic plants displayed an accelerated leaf senescence phenotype at the grain-filling stage [171] *NAM, ATAF1/2, and CUC2-FAMILY6*	± WUE Accelerates senescence; balance needed for net benefit
OsDREB 2B	LOC_Os05g27930	Involves in water- and heat-shock stress responses and tolerance [172] *DEHYDRATION-RESPONSE ELEMENT-BINDING PROTEIN2*	↑ WUE Boosts adaptive response under stress
OsbZIP23	LOC_Os02g52780	Overexpression shows improved tolerance to drought and high-salinity stresses and sensitivity to ABA [173] *BASIC-ZIPPER23*	↑ WUE Enhances tolerance through ABA sensitivity
CFL1	LOC_Os02g31140	Reduced expression resulted in the reinforcement of cuticle structure [174] *CURLY FLAG LEAF1*	↑ WUE Reduced expression improves barrier to water loss
OsCHR4	LOC_Os07g31450 LOC_Os07g32430	regulates leaf morphogenesis and cuticle wax formation [175] *CHROMATIN REMODELLING FACTOR4*	↑ WUE Enhances wax formation for water conservation
OsABA8 OX3	LOC_Os09g28390	Control ABA level and drought stress resistance in rice [176] *ABA 8´HYDROXYLASE3*	↑ WUE Enhances wax formation for water conservation
OsAPX7	LOC_Os04g35520	involved in signalling transduction pathways related to drought stress response [177] *ASCORBATE PEROXIDASE7*	↑ WUE Activates antioxidant pathways under stress

OsTPS1	LOC_Os01g23530<br>LOC_Os05g44210<br>LOC_Os05g44310<br>LOC_Os05g44300<br>LOC_Os08g34580	enhance the abiotic stress tolerance by increasing the amount of trehalose and proline [178]<br>*TREHALOSE-6-PHOSPHATE SYNTHASE1*	↑ WUE<br>Accumulates osmoprotectants for drought resilience

Supplementary References

149. International Rice Research Institute (IRRI), SNP-Seek Database. Available online: https://snp-seek.irri.org/ (accessed on 25 January 2024).
150. Yu, H., et al., Decreased photosynthesis in the erect panicle 3 (ep3) mutant of rice is associated with reduced stomatal conductance and attenuated guard cell development. Journal of Experimental Botany, 2015. 66(5): p. 1543-1552. https://doi.org/10.1093/jxb/eru525
151. Hwang, H., et al., Unique features of two potassium channels, OsKAT2 and OsKAT3, expressed in rice guard cells. PLoS One, 2013. 8(8): p. e72541. https://doi.org/10.1371/journal.pone.0072541
152. Moon, S.-J., et al., A dominant negative OsKAT2 mutant delays light-induced stomatal opening and improves drought tolerance without yield penalty in rice. Frontiers in Plant Science, 2017. 8: p. 772. https://doi.org/10.3389/fpls.2017.00772
153. Wu, Z., et al., Multiple transcriptional factors control stomata development in rice. New Phytologist, 2019. 223(1): p. 220-232. https://doi.org/10.1111/nph.15766
154. Chen, H., et al., Genetic bases of the stomata-related traits revealed by a genome-wide association analysis in rice (Oryza sativa L.). Frontiers in genetics, 2020. 11: p. 611. https://doi.org/10.3389/fgene.2020.00611
155. Nguyen, V.N., et al., OsABCG9 is an important ABC transporter of cuticular wax deposition in rice. Frontiers in Plant Science, 2018. 9: p. 960. https://doi.org/10.3389/fpls.2018.00960.
156. Islam, M.A., et al., Characterization of Glossy1-homologous genes in rice involved in leaf wax accumulation and drought resistance. Plant Mol Biol, 2009. 70(4): p. 443-56. https://doi.org/10.1007/s11103-009-9483-0
157. Wang, Y., et al., An ethylene response factor OsWR1 responsive to drought stress transcriptionally activates wax synthesis related genes and increases wax production in rice. Plant Mol Biol, 2012. 78(3): p. 275-88. https://doi.org/10.1007/s11103-011-9861-2
158. Sheng, Z., et al., Yellow-Leaf 1 encodes a magnesium-protoporphyrin IX monomethyl ester cyclase, involved in chlorophyll biosynthesis in rice (Oryza sativa L.). PLoS One, 2017. 12(5): p. e0177989. https://doi.org/10.1371/journal.pone.0177989
159. Li, C. and B. Zhang, MicroRNAs in control of plant development. Journal of cellular physiology, 2016. 231(2): p. 303-313. https://doi.org/10.1002/jcp.25125.
160. Wang, Y., et al., RICE CENTRORADIALIS 1, a TFL1-like Gene, Responses to Drought Stress and Regulates Rice Flowering Transition. Rice (N Y), 2020. 13(1): p. 70. https://doi.org/10.3389/fpls.2018.00960.
161. Li, M., et al., Mutations in the F‐box gene LARGER PANICLE improve the panicle architecture and enhance the grain yield in rice. Plant biotechnology journal, 2011. 9(9): p. 1002-1013. https://doi.org/10.1111/j.1467-7652.2011.00610.xhorizonepublishing.com+4
162. Sakamoto, T., Phytohormones and rice crop yield: strategies and opportunities for genetic improvement. Transgenic Res, 2006. 15(4): p. 399-404. https://doi.org/10.1007/s11248-006-0024-1
163. Ishiwata, A., et al., Two WUSCHEL-related homeobox genes, narrow leaf2 and narrow leaf3, control leaf width in rice. Plant Cell Physiol, 2013. 54(5): p. 779-92. https://doi.org/10.1093/pcp/pct032
164. Hu, J., et al., Identification and characterization of NARROW AND ROLLED LEAF 1, a novel gene regulating leaf morphology and plant architecture in rice. Plant Molecular Biology, 2010. 73(3): p. 283-292. https://doi.org/10.1007/s11103-010-9614-7
165. Sonah, H., et al., Molecular mapping of quantitative trait loci for flag leaf length and other agronomic traits in rice (Oryza sativa). Cereal Research Communications, 2012. 40(3): p. 362-372. https://doi.org/10.1556/CRC.40.2012.3.5
166. Miura, K., et al., OsSPL14 promotes panicle branching and higher grain productivity in rice. Nat Genet, 2010. 42(6): p. 545-9. https://doi.org/10.1038/ng.592
167. Hu, L., et al., OsSPL9 Regulates Grain Number and Grain Yield in Rice. Front Plant Sci, 2021. 12: p. 682018. https://doi.org/10.3389/fpls.2021.682018
168. Yu, D., et al., Wax Crystal-Sparse Leaf1 encodes a β–ketoacyl CoA synthase involved in biosynthesis of cuticular waxes on rice leaf. Planta, 2008. 228(4): p. 675-685. https://doi.org/10.1007/s00425-008-0770-9
169. Tang, Y., et al., Overexpression of a MYB Family Gene, OsMYB6, Increases Drought and Salinity Stress Tolerance in Transgenic Rice. Front Plant Sci, 2019. 10: p. 168. https://doi.org/10.3389/fpls.2019.00168

170. Sakurai, J., et al., Identification of 33 rice aquaporin genes and analysis of their expression and function. Plant and Cell Physiology, 2005. 46(9): p. 1568-1577. https://doi.org/10.1093/pcp/pci172
171. Zhou, Y., et al., Identification and functional characterization of a rice NAC gene involved in the regulation of leaf senescence. BMC Plant Biology, 2013. 13: p. 1-13. https://doi.org/10.1186/1471-2229-13-132
172. Matsukura, S., et al., Comprehensive analysis of rice DREB2-type genes that encode transcription factors involved in the expression of abiotic stress-responsive genes. Molecular Genetics and Genomics, 2010. 283: p. 185-196. https://doi.org/10.1007/s00438-009-0506-y
173. Xiang, Y., et al., Characterization of OsbZIP23 as a key player of the basic leucine zipper transcription factor family for conferring abscisic acid sensitivity and salinity and drought tolerance in rice. Plant physiology, 2008. 148(4): p. 1938-1952. https://doi.org/10.1104/pp.108.128199
174. Wu, R., et al., CFL1, a WW Domain Protein, Regulates Cuticle Development by Modulating the Function of HDG1, a Class IV Homeodomain Transcription Factor, in Rice and Arabidopsis The Plant Cell, 2011. 23(9): p. 3392-3411. https://doi.org/10.1105/tpc.111.088625link.springer.com+2
175. Guo, T., et al., Mutations in the rice OsCHR4 gene, encoding a CHD3 family chromatin remodeler, induce narrow and rolled leaves with increased cuticular wax. International Journal of Molecular Sciences, 2019. 20(10): p. 2567. https://doi.org/10.3390/ijms20102567
176. Cai, S., et al., A key ABA catabolic gene, OsABA8ox3, is involved in drought stress resistance in rice. PLoS One, 2015. 10(2): p. e0116646. https://doi.org/10.1371/journal.pone.0116646
177. Jardim-Messeder, D., et al., Stromal Ascorbate Peroxidase (OsAPX7) Modulates Drought Stress Tolerance in Rice (Oryza sativa). Antioxidants, 2023. 12(2): p. 387. https://doi.org/10.3390/antiox12020387
178. Li, H.-W., et al., Overexpression of the trehalose-6-phosphate synthase gene OsTPS1 enhances abiotic stress tolerance in rice. Planta, 2011. 234(5): p. 1007-1018. https://doi.org/10.1007/s00425-011-1458-0
